# A comprehensive approach to reduce sickness absenteeism

**DOI:** 10.5249/jivr.v7i1.631

**Published:** 2015-01

**Authors:** Saurabh RamBihariLal Shrivastava, Prateek Saurabh Shrivastava, Jegadeesh Ramasamy

**Affiliations:** ^*a*^Assistant Professor, Department of Community Medicine, Shri Sathya Sai Medical College & Research Institute, Kancheepuram, India.; ^*b*^Professor & Head, Department of Community Medicine, Shri Sathya Sai Medical College & Research Institute, Kancheepuram,India.

Globally, sickness absenteeism has gained a lot of public health significance in the last decade and is seen as an important determinant of productivity and overall development of the nation. ^1^ Sickness absenteeism is the absence of workers from the workplace due to illness / incapacity / other reasons, and the consequences of which clearly exceed the individual level (viz. loss of salary, increased medical expenses, poor quality of life, etc.) or the institutional level (viz. decrease production/quality of service, excess load on other colleagues, deteriorates relationship among the employer and employees, etc.) and eventually impacts the national / global economy. ^1,2^

Sickness absenteeism is not a true reflector of prevailing sickness in the work place as it is an extremely complicated phenomenon, with multi-faceted determinants such as medical causes; ^1^ economic causes (provided workers are entitled to sick leave with pay); ^1^ social causes (familial and social commitments); ^1^ work-related parameters (viz. employment in specific industries, workers' task/load, work shift, associated job stress, low hourly-wages, lack of satisfaction, minimal social support or co-workers support, and bullying/mistreatment at workplace); ^3-5^ administrator/ organization concerns (viz. poor commitment of the organization, absence of comprehensive policies to control sickness absenteeism, no measures for maintaining a healthy work environment, and compulsion to avail sanctioned leaves in a stipulated time frame); ^6^ non-occupational causes (viz. nutritional disorders, alcoholism and drug addiction); ^1,2^ and others (viz. female gender, older workers, full-time workers, negative work attitudes). ^2^

Owing to the global nature of the problem and significant impact on the life of people / nation’s economic progress, the need of the hour is to formulate a holistic policy, which necessitates the involvement of all stakeholders, and thus establish safe, healthy and more productive workplaces. ^2,3^ This comprehensive policy should consist of multiple strategies such as motivating administrators / employers for increasing organization commitment; ^6^ utilizing principles of ergonomics; ^1^ ensuring adequate pre-placement examination; ^1^ organizing periodical medical examinations to detect diseases at the earliest; ^1,2^ advocating use of personal protective measures at workplace; ^1^ offering health education and counseling services; ^1^ avoiding excessive workload on employees; ^3^ implementing measures to maintain healthy work environment and good human relations; ^2^ reducing job stress by encouraging participation of workers in recreational activities during their leisure time; ^2^ developing workplace mistreatment prevention strategies; ^5^ roping-in medical social workers to not only give social support but also encourage fast recovery of the worker; ^3^ and encouraging conduction of prospective or retrospective studies to understand the demographic and work characteristics so that customized and cost-effective strategies can be planned. ^3,6^

To conclude, the problem of sickness absenteeism is multi-factorial and complex, and thus there is an immense need to formulate a strategic policy to reduce economic expenditures, increase workers satisfaction, assist employers, and eventually facilitate economic development of the country.

## References

[1] Park K. Occupational health. In: Park K ( ed): Park’s Text Book of Preventive and Social Medicine. 20th ed. Jabalpur: Bhanot Publishers, 2009: 715.

[2] dos Santos K, Kupek E, Cunha JC, Blank VL (2011). Sickness-absenteeism, job demand-control model, and social support: a case-control study nested in a cohort of hospital workers, Santa Catarina, Brazil. Rev Bras Epidemiol.

[3] Ferreira RC, Griep RH, Fonseca Mde J, Rotenberg L (2012). A multifactorial approach to sickness absenteeism among nursing staff. Rev Saude Publica.

[4] Martimo KP (2006). Reducing sickness absenteeism at the workplace--what to do and how?. Scand J Work Environ Health.

[5] Asfaw AG, Chang CC, Ray TK (2014). Workplace mistreatment and sickness absenteeism from work: results from the 2010 National Health Interview survey. Am J Ind Med.

[6] Schalk R (2011). The influence of organizational commitment and health on sickness absenteeism: a longitudinal study. J Nurs Manag.

